# Xanthogranuloma of the External Auditory Canal Mimicking a Benign Tumor: A Case Report

**DOI:** 10.1155/2012/298089

**Published:** 2012-07-02

**Authors:** Keisuke Yoshihama, Yasumasa Kato, Yuh Baba

**Affiliations:** ^1^Department of Otolaryngology, Ohtawara Red Cross Hospital, 2-7-3 Sumiyoshi-cho, Ohtawara City, Tochigi 324-8686, Japan; ^2^Department of Oral Function and Molecular Biology, Ohu University, 31-1 Misumido, Tomita-machi, Koriyama City, Fukushima 963-8611, Japan; ^3^Department of Otolaryngology, Keio University, 35 Shinano-machi, Shinjuku, Tokyo 160-8582, Japan

## Abstract

Exostosis, osteoma, and adenoma are the most commonly encountered benign lesions in the external auditory canal. Herein, we report a case of the mass arising from the external auditory canal in a 24-year-old Japanese man. CT revealed the soft tissue mass without bony erosion, and MRI revealed that the mass showed a homogenous, iso signal intensity on a both T1- and T2-weighted image, suggesting that the mass is a benign tumor such as adenoma. Pathological examination showed that the specimen demonstrated xanthogranuloma in the external auditory canal. Although xanthogranuloma of the external auditory canal is extremely rare, otolaryngologists should recognize this condition during the inspection of the external auditory canal.

## 1. Introduction

Exostosis, osteoma, and adenoma are the most commonly encountered benign lesion in the external auditory canal. Xanthogranuloma is an uncommon non-Langerhans cell histiocytosis that usually occurs during infancy and early childhood. This lesion was first reported by Adamson in 1905 [1], who used the term “congenital xanthoma multiplex”. Helwig and Hackney subsequently introduced the term “juvenile xanthogranuloma” in 1954 [2]. Although similar lesions occur in adolescents and adults, the term “juvenile” is still commonly used. The most common sites of involvement are the head, neck, and trunk [3]. In recent years, however, it has become increasingly clear that there are numerous clinical forms of juvenile xanthogranuloma beyond this classic description. In such cases, the diagnosis may be more difficult to make without knowledge of the different possible clinical variants. We describe a case of juvenile xanthogranuloma with a rather unusual clinical presentation and highlight the importance of considering this entity in the differential diagnosis of benign soft tissue tumor and tumor-like lesion of the external auditory canal.

## 2. Case Report

**Figure 1:**
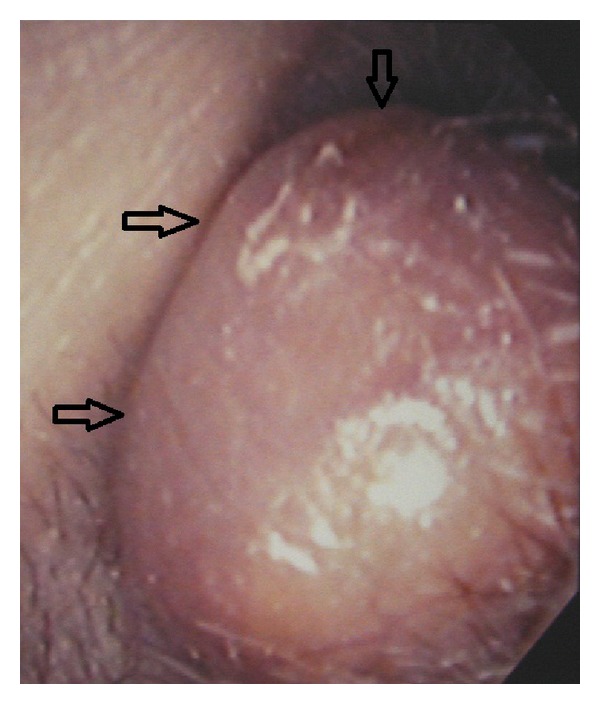
A smooth mass in the left external auditory canal (arrows).

**Figure 2:**
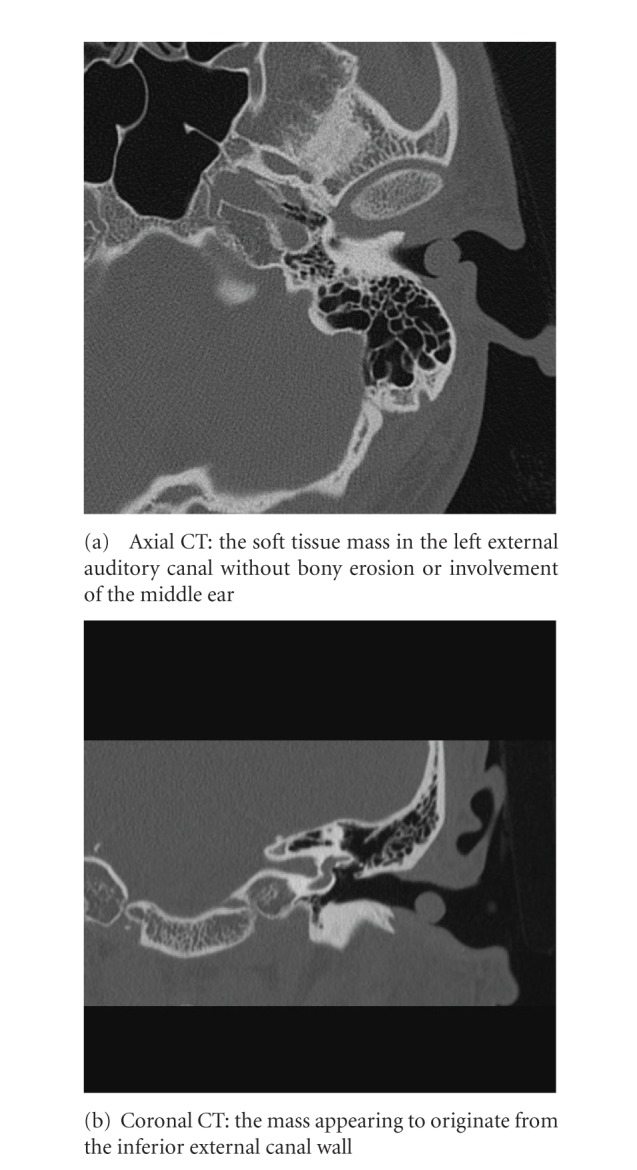
The CT findings.

**Figure 3:**
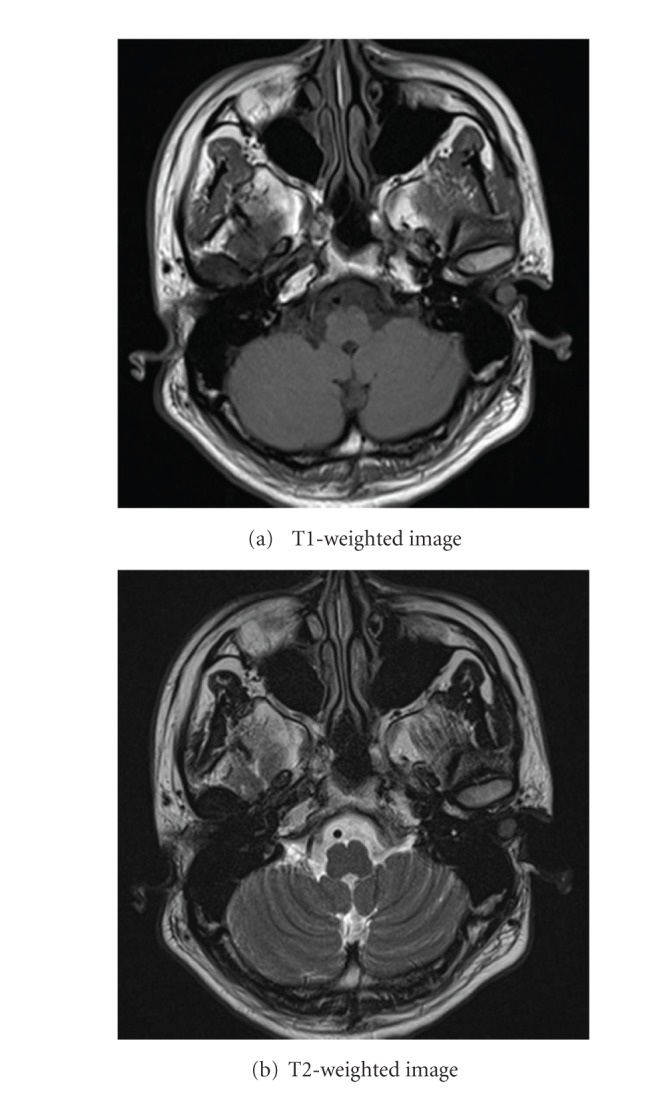
The MR findings: the mass measuring 10X10 mm showed a homogenous, iso signal intensity on a both T1- and T2-weighted image.

**Figure 4:**
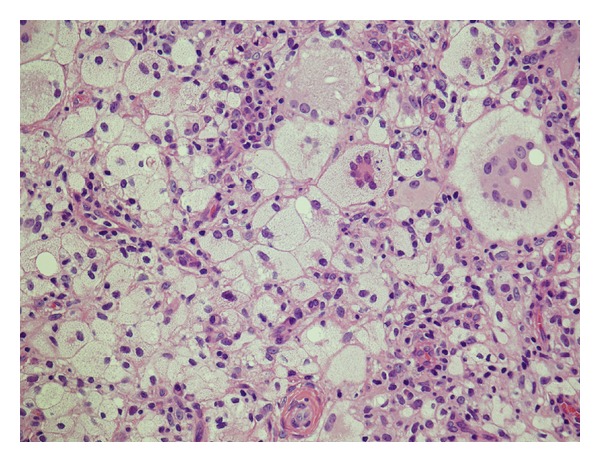
Microscopically, specimen demonstrated xanthogranuloma in the external auditory canal (hematoxylin and eosin, 100X).

Approximately one year ago, a 24-year-old Japanese man came for treatment with the complaint of intermittent left otalgia and blood-tinged otorrhea for 3 months. He noted a slight decrease in the hearing acuity of his left ear. He denied fever, vertigo, tinnitus, or facial weakness. He had no history of recurrent otitis media, trauma, or otologic surgery. Clinical examination revealed the presence of a mass that nearly occluded in the left external canal. The mass was smooth, elastic soft (Figure 1). The tympanic membrane appeared normal in fiberscope. No other skin or mucosal lesions were noted. Computed tomography scan (CT scan) revealed the soft tissue mass without bony erosion or involvement of the middle ear and appeared to originate from the inferior external canal wall (Figure 2). Magnetic resonance imaging (MRI) showed that the mass measuring 10 × 10 mm showed a homogenous, iso signal intensity on a both T1- and T2-weighted image (Figure 3). We performed cytological examination, and consequently it revealed no malignancy (data not shown). Blood test and the serum chemistry were within normal range. We considered it as a benign tumor which occurred from external canal wall. The patient underwent complete resection of the lesion through a transcanal approach, and we could easily extirpate it, because there was no adhesion between the mass and cartilage of ear canal. The histopathologic examination was that the predominant cell is the foamy histiocyte. The Touton giant cell is also commonly seen in this entity. This is a multinucleated cell with a peripheral ring of nuclei and a glassy, eosinophilic cytoplasm. This histopathologic examination is compatible with xanthogranuloma (Figure 4). A half year passed, and there was no recurrence. His left hearing after surgery is normal.

## 3. Discussion

Xanthogranuloma is a typical disease of non-Langerhans cell histiocytosis that comprises the group of diseases characterized in the proliferation of the histiocyte. Generally, it is known by the name of “juvenile xanthogranuloma”. It is chiefly generated in the head of infant multiple or solitary, but about 15% cases are generated in adults, from 20 to 40 years old is the peak age of onset. Although similar lesions occur in adolescents and adults, the term “juvenile” is still commonly used. The clinical behavior seems to differ depending on the age of the patient. The lesion occurring in infancy and childhood tends to be multiple and undergo spontaneous resolution within 1 year. In contrast the adolescent and adult form tends to be solitary and may persist. Our case was solitary type, because only one lesion was localized in the external ear canal. As our case was solitary type, it was difficult to differentiate xanthogranuloma from benign tumor. Although xanthogranuloma arising from the external auditory canal is extremely rare [4–6], we should consider this entity in the differential diagnosis of benign soft tissue tumor and tumor-like lesion of the external auditory canal.

The pathogenesis of xanthogranuloma is unknown, although the disease is believed to be a reactive rather than a neoplastic process. It is caused by the proliferation of plasmacytoid monocytes in response to an unknown etiologic agent, possibly either physical or infectious [7]. Because this patient often picks his ear, we postulate that it is caused in response to the habit of picking his ear.

 In summary, we describe an additional case of solitary xanthogranuloma involving the external auditory canal in this case.
